# Community Perceptions on Dementia-Friendly Infrastructure Needs: A Mixed-Method Study

**DOI:** 10.1093/geroni/igaf122.3901

**Published:** 2025-12-31

**Authors:** M D Sarafat Hossain, Sudikshya Sahoo, Nicole Ruggiano, Amie Brunson

**Affiliations:** University of Alabama, Tuscaloosa, Alabama, United States; University of Alabama, Tuscaloosa, Alabama, United States; University of Alabama, Tuscaloosa, Alabama, United States; University of Alabama, Tuscaloosa, Alabama, United States

## Abstract

Developing dementia-friendly communities with necessary infrastructural support can largely contribute to ensuring equitable access to care and public life among individuals with dementia. As part of the Building Our Largest Dementia Infrastructure (BOLD) initiative, this study investigated the perspectives of stakeholders (e.g., caregivers, providers, educators, NGO workers, Govt. employees) about the current infrastructural preparedness in Alabama and assesses the remaining needs to support dementia patients and their caregivers. Using a convergent mixed methods design, a statewide survey and series of focus groups were conducted to identify public priorities for supporting people with dementia, their families, and their providers. Quantitative data underwent uni- and multi-variate analyses and qualitative data underwent a multi-step coding process to identify themes. Data were also triangulated to enhance the trustworthiness of findings. A total of 29 people participated in four focus group discussion sessions and 397 people completed the online survey. The analysis identified four themes related to physical and social infrastructure that community members identified as priorities for improving dementia care: (1) infrastructural barriers to accessing information and care; (2) insufficient interventions to educate people about dementia; (3) unmet dementia caregivers’ needs for their social determinants of health; and (4) multi-system factors to improve the quality of dementia care. Survey data confirmed qualitative thematic findings. These findings have significant implications for how investments in practice, policy, and research may help close gaps in dementia care and promote dementia-friendly communities. Theoretically it reinforces the ecological system theory drawing attention to built and service environments for dementia.

